# Using relative handgrip strength to identify children at risk of sarcopenic obesity

**DOI:** 10.1371/journal.pone.0177006

**Published:** 2017-05-23

**Authors:** Michal Steffl, Jan Chrudimsky, James J. Tufano

**Affiliations:** Faculty of Physical Education and Sport, Charles University, Prague, Czech Republic; Ehime University Graduate School of Medicine, JAPAN

## Abstract

Identifying children at risk of developing childhood sarcopenic obesity often requires specialized equipment and costly testing procedures, so cheaper and quicker methods would be advantageous, especially in field-based settings. The purpose of this study was to determine the relationships between the muscle-to-fat ratio (MFR) and relative handgrip strength, and to determine the ability of handgrip strength relative to body mass index (grip-to-BMI) to identify children who are at risk of developing sarcopenic obesity. Grip-to-BMI was measured in 730 Czech children (4 to 14 yrs). Bioelectrical impedance was used to estimate body fat mass and skeletal muscle mass, from which the MFR was calculated.

The area under the curve (AUC) was 0.791 (95% CI 0.692–0.890, *p* ˂ 0.001) in girls 4–9; 0.789 (95% CI 0.688–0.890, *p* ˂ 0.001) in girls 10–14 years old; 0.719 (95% CI 0.607–0.831, *p* = 0.001) in boys 4–9; and 0.896 (95% CI 0.823–0.969, *p* ˂ 0.001) in boys 10–14 years old. Calculated using the grip-to-BMI ratio, the OR (95% CI) for girls to be at risk of sarcopenic obesity identified by MFR was 9.918 (4.243–23.186, *p* ˂ 0.001) and was 11.515 (4.280–30.982, *p* ˂ 0.001) for boys. The grip-to-BMI ratio can be used to predict the presence of sarcopenic obesity in children, which can play a role in pediatric health interventions.

## Introduction

The importance of monitoring health and fitness during the lifespan is well-known, especially when aiming to diagnose and treat diseases in their early stages. However, detailed health screening is oftentimes costly and may require technical equipment that is only available at medical facilities and administered by trained professionals. Therefore, implementing field-based fitness testing batteries provides an opportunity for the physical characteristics of large groups of individuals to be tested cheaply, efficiently, and simultaneously.

For example, it has been proposed that handgrip strength testing, among others, should be part of field-based test batteries for the assessment of physical fitness [1], as not only can handgrip strength be used to rapidly assess one’s general muscle strength [2], but it has been associated with numerous medical conditions across various age groups. Specifically, weak handgrip strength has been associated with an increased metabolic risk profile in children [3], has been linked to diabetes and other cardiometabolic risk factors in older adults [4], and has been connected with other parameters of physical fitness [5]. Additionally, handgrip strength can help predict the nutritional status of individuals [6] and can help identify individuals who suffer from malnutrition [7]. Perhaps most impactful, handgrip strength has been proposed as a tool for identifying neuromuscular disorders such as spinal muscular atrophy [8, 9], muscular dystrophy [10], and sarcopenia in the elderly [11].

As described by Rosenberg [12], sarcopenia is the age-related decrease in muscle mass and muscle strength that occur during the latter stages of aging. Although sarcopenia is a disease that is primarily associated with elderly populations, more recent research has shown that children may develop the condition as well [13]. Although children may lack muscle mass, the increasing presence of worldwide obesity presents another problem [14]. In children, it is not known whether a lack of muscle results in obesity or vice versa (i.e. the chicken or the egg phenomenon), but previous research has shown that obesity seems to contribute to the development of sarcopenia resulting in what is called “sarcopenic obesity” [15].

Sarcopenic obesity manifests when a disproportion exists between the amount of lean mass relative to fat mass [15, 16]. As sarcopenia is usually associated with a progressive decrease in muscle mass in the elderly [11, 17, 18], sarcopenic obesity likely better describes the disbalance between muscle and fat mass seen in children. Children may not outwardly appear to be obese, but may have a relatively low level of muscle mass compared to peers, which may be masked by a greater fat mass, resulting in a normal or healthy appearance. This makes it difficult to identify children who may have sarcopenic obesity. Therefore, a diagnostic tool for identifying sarcopenic obesity children may prove to be valuable, as neglecting treatment of any diseases in children may result in future health problems later in life.

Since children mature and develop at different rates, comparing absolute measurements of physical fitness may not be as appropriate as relative measurements, which can be applied across a variety of development stages in both genders. Therefore, McCarthy et al [19] suggested that the ratio of skeletal muscle mass (SMM) to body fat mass (BFM), creating a muscle-fat ratio (MFR), could serve as an indicator of metabolic risk in children. Initially, the MFR was proposed by Park et al [20] to determine the association between muscle mass and metabolic syndrome (MS), but McCarthy et al [19] took an extra step and proposed a method for calculating cut-off values in children using body mass index (BMI) together with MFR. Additionally Kim et al [21] proposed to use McCarthy’s method to identify children at risk of sarcopenia. Unfortunately, MFR relies on precise measurements of body composition (e.g. SMM and BFM), which are expensive to measure and cannot be measured in a timely manner when testing large groups of people. However, in clinical practice muscle strength, rather than muscle mass, may be used for diagnosing conditions such as sarcopenia.

Cruz-Jentoft et al [11] suggested using hand grip strength as an indicator of sarcopenia. Compared to tools such as dual energy x-ray absorptiometry (DXA) and bioelectrical impedance (BIA), handgrip strength can be measured quickly and easily in field-based testing. However, to account for changes in maturation and body size in children, grip strength should be expressed as a relative value. As a relative hand grip strength measure, the grip-to-BMI ratio has been proposed for diagnosing sarcopenia in the elderly [22]. Although a strong relationship between low lean mass and grip-to-BMI ratio has been declared in the elderly [22], there is limited information about such relationships in children.

Therefore, this study seeks to determine the relationships between MFR and grip-to-BMI ratio in the hopes that the grip-to-BMI ratio can prove to be an alternative to MFR for identifying children who may be at risk of developing sarcopenic obesity. Additionally, this study also aims to quantify the overall ability of the grip-to-BMI ratio to discriminate between children who are at risk of developing sarcopenic obesity and those who are not.

## Methods

### Participants

Children (n = 730, 4 to 14 yrs) of both genders participated in the current study. To include as many children as possible and represent as much of the child population as possible, there were no inclusion or exclusion criteria: all children that were physically able to participate took part in the study. Roughly 23% of children did not participate in any sport activities outside of school, 22% participated in various types of gymnastic activities (which is very popular in the Czech Republic), 31% participated in sport games such as soccer and ice hockey, and 25% participated in other sports such as karate, judo, athletics, and other individual sports outside of school. Additionally, 23% of children spent no time doing sport outside of school, about 23% reported doing less than 4 hours of sport per week, and about 54% participated in sport outside of school over 4 hours per week. All children were recruited from six cities across the Czech Republic (Prague, Brno, Ceske Budejovice, Plzen, Zlin, and Zelenec) during a sport and active lifestyle promotional event series called Sportacek 2015, which was conducted throughout 2015 in association with the Faculty of Physical Education and Sport at Charles University in Prague. The study was carried out with approval from the faculty’s ethics committee and legal guardians provided written informed consent for all children.

### Outcome measures

While fully clothed and no shoes or socks, body height was measured using a SECA 213 portable stadiometer, body mass was measured using a SECA 876 digital flat floor scale, and body composition was measured via bioelectrical impedance (InBody 720, Biospace Co., Ltd. Korea). From the InBody device, SMM and BFM were obtained. To calculate MFR, SMM was divided by BFM. Using a hand grip dynamometer (Takei A5401), seated grip strength was measured for the right and left hands independently using standardized procedures with the humerus positioned at the side of the body and the elbow flexed to 90 degrees [23]. For each trial, subjects were instructed to squeeze the dynamometer with maximal effort for two to three seconds. Participants performed three successive trails for each hand with a few seconds of rest between each trial. The handgrip strength of three trials for the right and left hands was measured, and the strongest record from those three measurements was recorded for each, the right and left hands. Then, the best value, whether from the right or left hand, was used as the maximal handgrip strength value. By dividing maximal handgrip strength by BMI, the grip-to-BMI ratio was calculated. All data is available in S1 Dataset.

### Sarcopenia risk diagnostics

According to previous methodology used to define sarcopenia in children described by McCarthy et al [19] and Kim et al [21], girls and boys were divided into two age ranges: 4–9 years and 10–14 years. Then, they were divided into quintiles of BMI z-scores and a cut-off value for sarcopenic obesity diagnostics was defined as 2SD lower than the mean MFR for the 3^rd^ BMI quintile. This was done for each age range and both genders, separately.

### Statistics and data analysis

First, the data were tested for normality using the Kolmogorov-Smirnov test. Since the majority of data were not normally distributed, medians and interquartile ranges (IQR) for all variables were calculated for each gender separately. Differences between genders were tested using the Two-Sample Kolmogorov-Smirnov test for continuous variables and the Pearson Chi-Square test for categorical variables. Second, descriptive statistics of the preferred hand were calculated for age groups according to genders. Third, receiver-operating characteristic curves (ROC) were used to determine the ability of grip-to-BMI ratio to estimate the risk of sarcopenic obesity in children. In the ROC curves, the AUC was calculated and can be interpreted as follows: 0.9–1.0 excellent; 0.8–0.9 good; 0.7–0.8 fair; 0.6–0.7 poor, and 0.5–0.6 fail (*p* ˂0.05) [24]. Fourth, ROC analysis cut-off points of grip-to-BMI ratio to identify sarcopenic obesity were calculated for each gender and each age category separately. The best cut-off point for balancing the sensitivity and specificity of the test was defined as that yielding the minimal value for the equation = (1-sensitivity)^2^ + (1-specificity)^2^ [24]. Fifth, the age-adjusted binary logistic regression model for each gender separately was used to estimate the odds of developing sarcopenic obesity according to MFR when a subject was at risk of sarcopenic obesity according to cut-off values in grip-to-BMI. Effect sizes are reported by odds ratios (ORs; i.e., exponents of the estimates). Statistical calculations were carried out in IBM SPSS Statistics 22.

## Results

**Fig 1:**
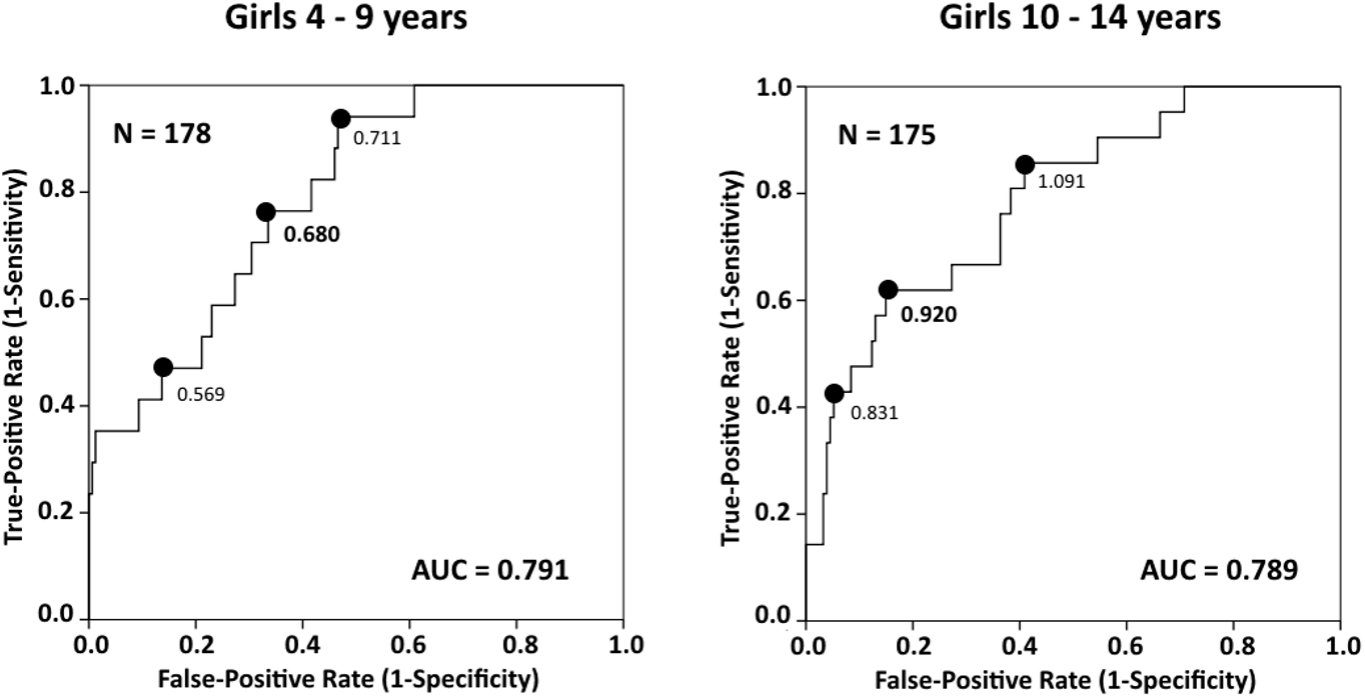
Receiver operating characteristic (ROC) curves for identifying sarcopenic obesity according to different cut points for grip-to-BMI (kg/kg) in girls.

**Fig 2:**
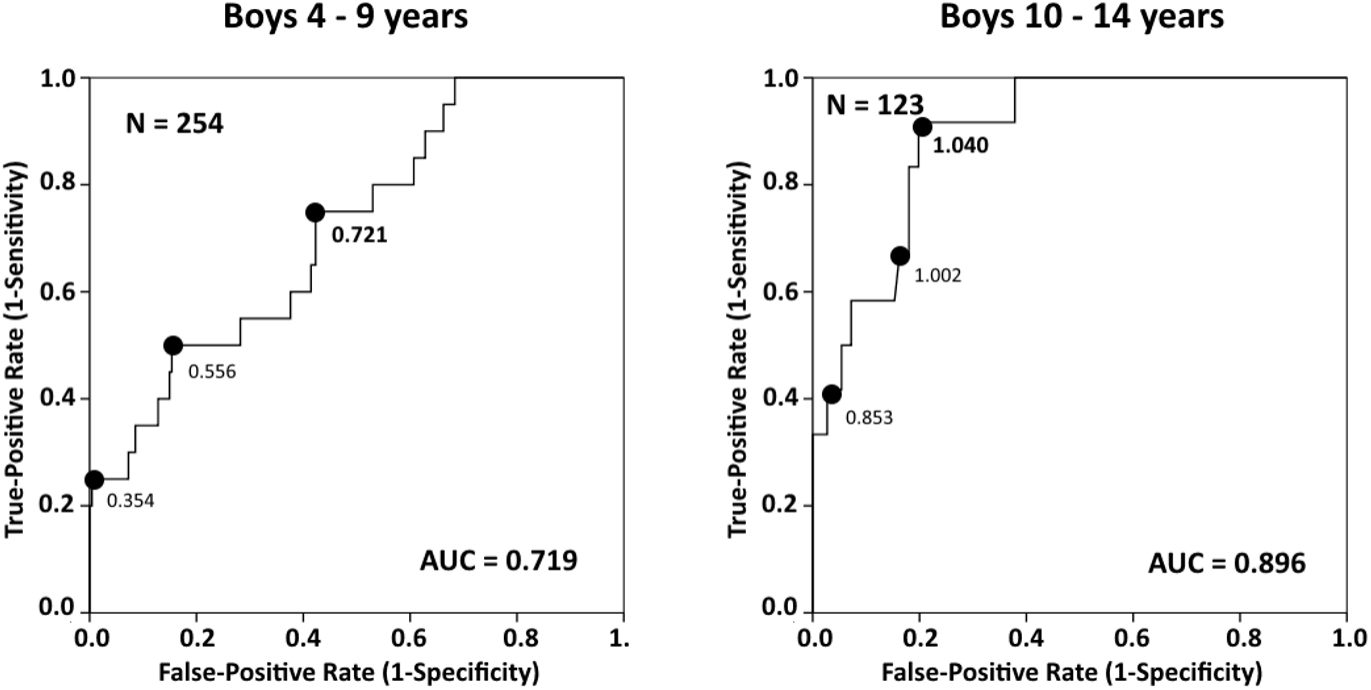
Receiver operating characteristic (ROC) curves for identifying sarcopenic obesity according to different cut points for grip-to-BMI (kg/kg) in boys.

Of the 730 total children, 353 were girls, median age 9 (IQR 4), and 377 were boys, median age 8 (IQR 4). The majority of children were from Prague (29.9%) and Zelenec (26.8%). The remainder of the children came from Zlin, 13.2%; Plzen, 11.9%; Brno, 10.8%; and Ceske Budejovice, 7.4%. Descriptive statistics are presented in Table 1. The genders were significantly different in terms of age, height, weight, BMI, SMM, body-fat percentage (BFP), BFM, and MFR (Table 1). Detailed results of handgrip strength and preferred side are presented according to separate age categories in Table 2. The cut-off value for MFR using the 3^rd^ BMI quintile was 1.22 (kg/kg) for girls and 1.35 (kg/kg) for boys. The AUC was 0.791 (95% CI 0.692–0.890, *p* ˂ 0.001) in girls 4–9; 0.789 (95% CI 0.688–0.890, *p* ˂ 0.001) in girls 10–14 years old; 0.719 (95% CI 0.607–0.831, *p* = 0.001) in boys 4–9; and 0.896 (95% CI 0.823–0.969, *p* ˂ 0.001) in boys 10–14 years old. The cut-off point was estimated as 0.680 kg/kg for girls 4–9, 0.920 kg/kg for girls 10–14, 0.721 kg/kg for boys 4–9, and 1.040 kg/kg for boys 10–14 years old. The estimation of the optimal cut-off point is presented in **Table 3**. The ROC curves for girls are shown in Fig 1 and in Fig 2 for boys. According to the age-adjusted binary regression model, the OR (95% CI) was 9.918 (4.243–23.186, *p* ˂ 0.001) in girls and 11.515 (4.280–30.982, *p* ˂ 0.001) in boys.

**Table 1:** Descriptive statistics of boys and girls.

	Girls	Boys	*p* value
*N* (%)	353 (48.4)	377 (51.6)	0.374
Age (years)	9 (4)	8 (4)	<0.001
Height (cm)	140 (24)	133 (22)	<0.001
Weight (kg)	32.2 (16.4)	28.6 (12.0)	0.001
BMI (kg/m^2^)	16.8 (3.5)	16.5 (2.5)	0.015
SMM (kg)	13.9 (7.1)	13.1 (6.3)	0.049
BFP (%)	16.9 (9.0)	12.6 (8.1)	<0.001
BFM (kg)	5.5 (5.1)	3.5 (3.6)	<0.001
MFR (kg/kg)	2.5 (1.6)	3.6 (2.7)	<0.001
Handgrip (kg)	15.1 (9.0)	14.7 (8.7)	0.344
Grip-to-BMI (kg/kg/m^2^)	0.90 (0.4)	0.87 (0.4)	0.829
Sarcopenic obesity by MFR *n* (%)	33 (9.3)	27 (7.2)	0.283

BMI = body mass index; SMM = skeletal muscle mass; BFM = body fat mass; MFR = muscle fat ratio, Two-Sample Kolmogorov-Smirnov test for continuous variables and the Pearson Chi-Square test for categorical variables.

**Table 2:** Overview of handgrip strength data.

	Boys						
	Right-preference				Left-preference		
Age	*N* (%)	Left hand (kg)	Right hand (kg)		*N* (%)	Left hand (kg)	Right hand (kg)
4–9	167 (65.7)	10.9 (3.5)	12.4 (3.7)		87 (34.3)	12.0 (3.9)	10.8 (3.7)
10–14	88 (71.5)	21.7 (8.0)	24.0 (8.7)		35 (28.5)	22.4 (6.6)	20.4 (6.3)
	Girls						
	Right-preference				Left-preference		
Age	*N* (%)	Left hand (kg)	Right hand (kg)		*N* (%)	Left hand (kg)	Right hand (kg)
4–9	121 (68.0)	10.4 (3.4)	11.7 (3.4)		57 (32.0)	11.3 (3.9)	9.8 (3.8)
10–14	124 (70.9)	19.2 (4.9)	21.6 (5.3)		51 (29.1)	20.4 (5.8)	18.7 (5.9)

Data are presented as mean (SD).

**Table 3:** Estimation of optimal cut-off point of grip-to-BMI ratio.

		%		
	Cut-off point	Sensitivity	Specificity	Equation
Girls 4–9	0.569	52.9	78.9	0.266
	**0.680**	76.5	60.2	0.214
	0.711	94.1	53.4	0.221
Girls 10–14	0.831	42.9	94.8	0.329
	**0.920**	61.9	85.1	0.167
	1.091	81.0	59.1	0.203
Boys 4–9	0.354	25.0	99.6	0.563
	0.556	50.0	84.6	0.274
	**0.721**	75.0	57.7	0.241
Boys 10–14	0.853	41.7	97.3	0.341
	1.002	66.7	83.8	0.137
	**1.040**	91.7	80.2	0.046

Equation = (1—Sensitivity)^2^ + (1—Specificity)^2^

## Discussion

Previous researchers have determined that body composition analyses can allow for the identification of those who may be diagnosed with sarcopenic obesity [15]. However, direct assessment of body composition is costly and the ability to cheaply and quickly estimate specific aspects of body composition in children (e.g. low SMM) would prove to be valuable. The data of the present study show that by simply using grip strength and BMI measurements, the grip-to-BMI ratio can serve as a tool for identifying children who are at risk of being diagnosed with sarcopenic obesity.

Although sarcopenia has traditionally been associated with the loss of muscle mass in the elderly, recent evidence shows that inactive children may develop sarcopenia as well [21]. In contrast to the elderly, where sarcopenia is a single component of a combination of degenerative processes, the reason why children are at risk of developing sarcopenia is quite different. It is probable that obesity, induced by a lack of physical activity, plays a role in developing childhood sarcopenia, as the prevalence of childhood obesity is increasing worldwide [14, 25]. As body fat increases and the MFR decreases, in favor of body fat, relative muscle strength likely decreases. Therefore, it is possible that measuring relative muscle strength would be a logical alternative to costly body composition measurements when identifying children at risk of sarcopenic obesity.

Although associations between muscle strength and child sarcopenic obesity, strength has been associated with sarcopenia in the elderly. Specifically, weak handgrip strength has been considered to be a better indicator for diagnosing sarcopenia in the elderly than low muscle mass [26]. Additionally, handgrip strength [11] and the grip-to-BMI ratio [22] have been used to clinically assess sarcopenia in the elderly. However, in children, even though a strong correlation exists among weight, height, and handgrip strength [27, 28], there is a lack of information regarding the relationship between handgrip strength, fat mass, and muscle mass.

In children, the MFR has been considered as the main indicator of low muscle mass [21]. Unfortunately, MFR calculations depend on anthropometric measures and body fat estimations that require advanced assessments using specialized equipment such as DXA or BIA. However, handgrip strength measurements are relatively cheaper and easily applied. When expressed as a relative value, the grip-to-BMI ratio was able to discriminate between children at risk versus those who are not. Children whose grip-to-BMI ratio was considered as low had quite high odds of being diagnosed at risk of sarcopenic obesity by MFR. According to the standard interpretation of AUC, grip-to-BMI provides a fair accurate estimation.

The present study contains some limitations that are worth mentioning. First, the children included in the sample were recruited from an active lifestyle promotional event series, meaning that the children and their families may not represent the entirety of the Czech population. Rather, the children included in the study may have been more active than their sedentary peers, possibly resulting in a population-specific pool of “stronger than normal” children, but such data was not collected. Second, balancing the best cut off point is usually difficult, because any increase in sensitivity will be accompanied by a decrease in specificity. We applied the method which was recommended in such a case [24] and finally the false positivity was 0.398 in girls 4–9, 0.149 in in girls 10–14, 0.423 in boys 4–9, and 0.198 in boys 10–14 which is quite high however, still it is acceptable.

However, the prevalence of sarcopenic obesity risk in our study was higher in both genders (8%) compared to a previous study that showed only 0.1% of Korean boys and 3.8% of Korean girls were at risk of sarcopenia class II; however if the authors had calculated sarcopenia class I, as 1 SD lower than the mean MFR for the 3^rd^ BMI quintile, the prevalence would have been greater: 32.1% in boys and 24.3% in girls [21]. Additionally, the authors used appendicular skeletal muscle mass measured by DXA, whereas we used BIA. In another study where the cut-off points for MFR were similar (1.25 for boys in both age groups and 1.10 in the younger girls and 0.80 for older girls), the prevalence was 9% in boys and 9.8% in girls [19], which are similar to the values in the present study. Additionally, although BIA has been shown to be a valid and reliable tool for assessing body composition [29], such systems are not capable of direct measurements and simply estimate body composition via electric signal transmission through the body, calculated using a set of normative anthropometric data. Therefore, it is possible that a more direct measurement of body composition such as DXA may have provided more accurate data. On the same token, although the MFR calculated using BIA provides information regarding the amount of skeletal muscle mass within the body, it is not possible for BIA to determine the root cause of the MFR (i.e. changes to the MFR could be caused by malnutrition, physical inactivity, chronic inflammation, myopathy, etc.). Therefore, any measurement using MFR should not be used to clinically diagnose sarcopenic obesity in children, but instead to provide a quick, valid, and reliable “first-glance” into children’s body composition, identifying those who may warrant a more detailed examination.

In agreement with the hypothesis, the main finding of the present study was that the grip-to-BMI ratio was able to discriminate between children who may be diagnosed with sarcopenic obesity and those who may not, and could serve as a good field-based method. Future research should aim to confirm these findings using samples from other populations. Although the methods proposed in this study cannot directly determine the presence of sarcopenic obesity in children, these measurements can serve as a cheap and efficient method of identifying those who may be at risk and who may need more detailed medical examinations, nutritional interventions, or exercise prescriptions.

## Supporting information

S1 Dataset (XLSX)
